# Immigrant and non-immigrant women’s experiences of maternity care: a systematic and comparative review of studies in five countries

**DOI:** 10.1186/1471-2393-14-152

**Published:** 2014-04-29

**Authors:** Rhonda Small, Carolyn Roth, Manjri Raval, Touran Shafiei, Dineke Korfker, Maureen Heaman, Christine McCourt, Anita Gagnon

**Affiliations:** 1 Judith Lumley Centre, La Trobe University, 215 Franklin Street, Melbourne VIC 3000, Australia; 2 Clinical Education Centre, Keele University, Newcastle Road, Staffordshire ST4 6QG, UK; 3 TNO Institute, Wassenaarseweg, Leiden CE 56 2301, Netherlands; 4 Faculty of Nursing, Helen Glass Centre for Nursing, 89 Curry Place, University of Manitoba, Winnipeg, MB R3T 2N2, Canada; 5 School of Health Sciences, City University London, Bartholomew Close, London EC1A 7QN, UK; 6 McGill, Ingram School of Nursing & Department Ob/Gyn, MUHC Prog.Ob/Gyn, 3506 rue University, Montreal, Quebec H3A 2A7, Canada

**Keywords:** Maternity care, Immigrant women, Experiences of care, Communication

## Abstract

**Background:**

Understanding immigrant women’s experiences of maternity care is critical if receiving country care systems are to respond appropriately to increasing global migration. This systematic review aimed to compare what we know about immigrant and non-immigrant women’s experiences of maternity care.

**Methods:**

Medline, CINAHL, Health Star, Embase and PsychInfo were searched for the period 1989–2012. First, we retrieved population-based studies of women’s experiences of maternity care (n = 12). For countries with identified population studies, studies focused specifically on immigrant women’s experiences of care were also retrieved (n = 22). For all included studies, we extracted available data on experiences of care and undertook a descriptive comparison.

**Results:**

What immigrant and non-immigrant women want from maternity care proved similar: safe, high quality, attentive and individualised care, with adequate information and support. Immigrant women were less positive about their care than non-immigrant women. Communication problems and lack of familiarity with care systems impacted negatively on immigrant women’s experiences, as did perceptions of discrimination and care which was not kind or respectful.

**Conclusion:**

Few differences were found in what immigrant and non-immigrant women want from maternity care. The challenge for health systems is to address the barriers immigrant women face by improving communication, increasing women’s understanding of care provision and reducing discrimination.

## Background

Increasing global migration has implications both for health care provision in receiving countries and for the health care experiences of immigrant populations. This is nowhere more apparent than in the experience of women giving birth post-migration. A systematic review of immigrant women’s perinatal outcomes published in 2010 [1] identified very few studies over a ten-year period which described any aspect of immigrant women’s maternity care experiences in comparison with non-immigrant women. Some population-based studies of women’s experiences of maternity care conducted in a few countries do include limited data on immigrant and refugee women’s experiences of care for comparison with non-immigrant women, but immigrant women are commonly under-represented in these studies because of the formidable challenges of undertaking inclusive cross-cultural research that is population-based and large scale [2,3]. These challenges include: sampling and recruitment issues, difficulties in translation and in assessment of validity with the use of standard research instruments, and increased research costs. Other studies have specifically investigated the experiences of individual groups of immigrant and refugee women, and to date these are mostly small and qualitative. Given the dearth of adequately-sized and appropriately conducted studies directly comparing representative immigrant and non-immigrant experiences of maternity care, a systematic review drawing on data in general population studies and in specific immigrant studies in the same countries, would seem to offer the best opportunity for drawing together and comparing what is known about immigrant and non-immigrant experiences, and what women want – and get – from their maternity care.

Our purpose in selecting studies for this review was thus twofold. First, we aimed to identify and review all published population-based studies of women’s experiences of maternity care to determine what they say about what women want from care, including any data, if available, about immigrant women. Second, having identified the countries where such studies have been conducted, we aimed to investigate further what is known about the experiences of immigrant women in each of these countries, by identifying and reviewing studies focused specifically on immigrant women’s experiences of their maternity care. For the purposes of this review, we define immigrant women as those women not themselves born in the country in which they are giving birth.

There were two review questions:

1. What do immigrant and non-immigrant women want from their maternity care?

2. How do immigrant and non-immigrant women’s experiences and ratings of care compare, both within and across included countries?

## Methods

### Search strategy

Ovid was used to search the electronic databases Medline, CINAHL, Health Star, Embase and PsychInfo for the period 1989–2011. The search strategy was developed by MR with the assistance of the Health Sciences Librarian at La Trobe University in February 2010 and further searches were conducted to update the literature to December 2012. 1989 was chosen as the start year because the first population-based study of women’s experiences of maternity care was known to have been conducted in that year [4]. Terms combined in the search included: emigration/emigrant, immigration/immigrant, migrant, ethnic group, ethnic minority, population groups, refugees, non-English speaking, women, view, opinion, attitude, experience, maternal health services, maternity care, perinatal care, prenatal/antenatal care, intrapartum care, postnatal care, delivery, obstetrics, midwifery. For an example of the search strategies used, see Additional file 1.

### Inclusion and exclusion criteria

Population-based studies of women’s experiences of care, defined as those with national or regional samples with representativeness assessed, were identified, retrieved and reviewed. Studies with a hospital-based or convenience sample or where representativeness could not be assessed were excluded. With these criteria, 12 studies from five countries were included [4-24]. One national study was identified from Scotland, [25] but subsequently excluded, as its overall population representativeness could not be assessed.

Studies focusing specifically on immigrant women’s experiences of maternity care from these same five countries were then also identified, retrieved and reviewed. Studies of ethnic minorities who were not themselves immigrants or refugees were excluded, as were retrieved studies which on review, were found to focus only on cultural beliefs and practices around childbirth without investigating immigrant women’s actual experiences of the maternity care they received. For the immigrant studies, all retrieved studies were included (i.e. no quality criteria were applied), for two reasons. First, our purpose was to include as much data as possible about a diverse range of immigrant women’s experiences for comparison with data on non-immigrant women from the population-based studies. Second, the immigrant studies were relatively few across the included countries; and most were small and qualitative. Twenty-two studies of immigrant women’s experiences of care were identified, retrieved and reviewed across the five included countries [26-55].

### Approach to analysis

Papers were read and the findings summarised, noting (where available) overall ratings of care and key conclusions about what women wanted from care (RS, MR and TS). The country, year of study, sample size and study type (e.g., population-based postal survey, qualitative interview study) were also noted. For the population-based studies, the main findings were recorded separately for non-immigrant and immigrant women, except when the data did not distinguish these groups of interest (the three US studies and two of the UK studies). Study findings were tabulated for ease of discussion and interpretation (MR and RS) and a descriptive thematic analysis of the extracted data was undertaken [56]. Two authors independently developed codes for describing the data (MR and RS) and a third author (TS) reviewed these. The resulting interpretation of the data was then reviewed and revised by all authors.

## Results and discussion

**Figure 1:**
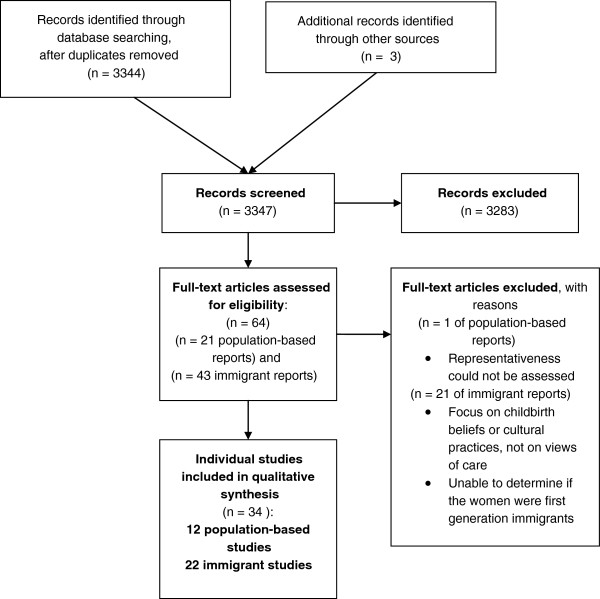
Flow diagram of the review process and selection of studies.

Figure 1 provides a flow diagram of the review process and the selection of studies.

### The countries and the included studies

#### Australia

Three population-based studies from the state of Victoria (1989, 1994, 2000) [4-10] and seven studies of immigrant women (including Vietnamese, Chinese, Cambodian, Laotian, Thai, Korean, Filipino, Turkish, Muslim women from a range of countries) [26-39] were reviewed.

#### Canada

One national survey (2006) [11,12] and four studies of immigrant women (including Somali, South Asian, Punjabi, Muslim women from various countries) [40,43] were reviewed.

#### Sweden

One national study (1999–2000) [13-15] and two studies of immigrant women (including immigrant Somali, Eritrean and Sudanese women) [44,45] were reviewed.

#### United Kingdom

Four national surveys (1995, 2006 and 2007) [16-19] and six studies of immigrant women (including immigrant South Asian, Somali, Indian, Pakistani and Bangladeshi women) [46-51] were reviewed.

#### USA

Three national surveys (2002, 2006 and 2013) [20-24] and four studies of immigrant women (including Somali, Hmong, Puerto Rican and ‘Hispanic’ immigrant women) [52-55] were reviewed. Although Puerto Rico is an unincorporated US Territory, not a separate country, Puerto Rican women coming to the US have been considered ‘immigrants’ for the purposes of this review.

These 12 population-based studies from five countries were conducted in the period 1989–2013 and involved 55,495 women (range 790–26,325). In four of the studies [16,18,20,22] (involving 31,887 women), it was not possible to determine women’s country of birth in order to calculate the number of women who were immigrants. For the remaining eight studies [4-15,17,19] (involving 23,608 women) there were 2,682 women (8.3%) who were immigrants and 15,593 women who were non-immigrants. For the 22 specific studies of immigrant women [26-55], sample sizes ranged from 6 to 432, with a total of 2,498 immigrant women involved, with studies published between 1990 and 2012.

### What do non-immigrant women want from their maternity care?

The key findings from the population-based studies about what non-immigrant women appreciate and want from their maternity care proved remarkably similar across the included countries, as can be seen in the study summaries provided in Table 1. Most of these population-based studies assessed women’s overall ratings of care for each of the three phases of care: during pregnancy, during labour and birth and during the postpartum hospital stay. The exceptions to this were: the Canadian survey, in which women were asked to rate their satisfaction with six aspects of their interaction with health care providers during the entire pregnancy, labour and birth, and immediate postpartum period, [11] and the US surveys, where women were not asked to give overall ratings of their care except in response to a question in the 2005 and 2013 surveys asking women their view about the maternity care system overall, with 35% and 36% rating it as excellent, 47% and 47% as good, and 16% and 17% as poor, respectively [22,24].

**Table 1:** Population-based studies of women’s experiences of maternity care

***AUSTRALIA***		
**Survey of Recent Mothers in Victoria 1989**[4,5]		
n=790, including 92 immigrant women from non-English speaking (NES) countries		
Postal survey, one week of births.		
Overall: 88% rated antenatal care as very good/good, 67% said care in labour and birth was managed as they liked.		
NES-immigrant women: 72% rated antenatal care as very good/good		
**Survey of Recent Mothers in Victoria 1994**[6,7]		
n=1336; including 142 immigrant women from non-English speaking (NES) countries.		
Postal survey, two weeks of births.		
Overall: 63% rated antenatal care as very good, 71% for care in labour and birth, and 52% for postnatal hospital care.		
NES-immigrant women: 45% rated antenatal care as very good, 42% for care in labour and birth, and 40% for postnatal hospital care		
**Survey of Recent Mothers in Victoria 2000**[8-10]		
n=1616; including 164 immigrant women from non-English speaking (NES) countries		
Postal survey, two weeks of births.		
Overall: 67% rated antenatal care as very good, 72% for care in labour and birth, and 51% for postnatal hospital care.		
NES-immigrant women: 49% rated antenatal care as very good, 55% for care in labour and birth, and 40% for postnatal hospital care		
**Overall findings about what women want: all three surveys**	**Key findings for immigrant women: all three surveys**	**Conclusions and key recommendations: all three surveys**
Adequate information and explanations, concerns addressed	Immigrant women were under-represented in all three surveys, nevertheless:	Access to information, good relationships with caregivers and involvement in decision making were critical to enhancing women’s positive ratings of their care
Active say in decisions about care		
Caregivers being helpful, not rushed, sensitive, kind and understanding	What immigrant women wanted was very similar to the overall findings, including: good explanations, an active say in decisions, helpful, kind caregivers and support with infant care after birth	Recommendations include:
Knowing caregivers (eg knowing midwife before labour, birth centres, own doctor; knowing midwives on postnatal ward)	Women born overseas in non-English speaking countries were less positive about their maternity care than women born in Australia or than women born overseas in English speaking countries	Greater focus on continuity of care provision, improving staff communication and listening skills and more woman-centred, individualised care
Receiving helpful, consistent and supportive advice about infant feeding and care		
***CANADA***		
**Maternity Experiences Survey (MES) 2006**[11,12]		
n=6421; including 470 recent immigrants.		
Computer Assisted Telephone Interviews (CATIs) in French, English and 13 community languages. Sample drawn from Canadian Census.		
Overall: 54% rated their overall experience of labour and birth as “very positive”;		
79% felt they were shown respect; and 73% were happy with their participation in decision-making.		
**Overall findings about what women want**	**Key findings for immigrant women**	**Conclusions and key recommendations**
Little data about factors contributing to satisfaction with care and what women wanted and valued.	Despite interviews conducted in English, French and 13 community languages, women reporting a first language other than English or French, were under-represented.	Recommendations not specifically focused on potential improvements to care based on women’s experiences. Rather recommendations focused on the need for more education for caregivers and women about evidence-based care practices (eg need to reduce the extent of routine use of electronic fetal monitoring and episiotomy, and supine position for birth).
Women with a midwife as the primary birth attendant and those with no interventions in labour were more satisfied with care.	17% of recent immigrant women reported not receiving care in a language they could understand.	
Half the women thought having the same care provider for pregnancy, labour and birth was important.	No differences reported between groups (i.e., recent immigrants, non-recent immigrants, and Canadian-born women) in their satisfaction with the compassion, competence, privacy, or respect demonstrated by their health care provider or their own involvement in decision-making during the entire pregnancy, labour and birth, and immediate postpartum period [9].	For immigrant women, recommendations focused on the need for education about improving health behaviors such as pre-conception use of folic acid, screening for postpartum depression, improving access to health care providers in the postpartum period, and removing language barriers to seeking care.
***SWEDEN***		
**National cohort study of women’s experiences of childbirth (KUB) 1999-2000**[13-15]		
n=2746; 266 immigrant women		
Postal survey		
Overall: 53% very positive about intrapartum care and 35% about postpartum care		
**Overall findings about what women want**	**Key findings for immigrant women**	**Conclusions and key recommendations**
Caregivers who provide adequate support and information, with enough time to answer questions and give help; and who are friendly, non-judgemental and respectful	Non-Swedish speaking women were excluded, nevertheless: women born outside Sweden were somewhat less happy with their care than Swedish-born women:	Authors recommend midwives support patients in a professional and caring manner, asking women about their needs for information and offering individualised care.
Continuity of care: small numbers of care providers preferred Attention paid to partners’ needs		Acknowledgement that non-Swedish speaking women were excluded, thus those foreign-born women recruited were likely to be more integrated into Swedish society.
Pre-birth visits to labour ward		
***UNITED KINGDOM***		
**First class delivery: A national survey of women’s views of maternity care 1995**[16]		
n=2406; numbers of immigrant women not reported		
Postal survey		
**Recorded delivery: A national survey of women’s experiences of maternity care**[17]**2006**		
n=2966; 229 black and ethnic minority women born outside UK		
Postal survey		
Overall: 48% very satisfied with antenatal care; 56% with care for labor and birth; and 39% with postnatal care		
**Towards Better Births: a survey of recent mothers 2007**[18]		
n=26,325; numbers of immigrant women not reported		
Postal survey, sample drawn from NHS Trusts in England		
Overall: 68% rated antenatal care as excellent or very good; 75% for care in labor and birth; and 69% for postnatal care		
**Delivered with care: a national survey of women’s experiences of maternity care 2012**[19]		
n=5,333; 1,152 immigrant women		
Postal or online survey:4,945 postal respondents; 407 online respondents		
Overall: 88% very satisfied or satisfied with antenatal care; 87% with care for labor and birth; 76% with postnatal care		
**Overall findings about what women want: all four surveys**	**Key findings for immigrant women: two surveys**	**Conclusions and key recommendations: all four surveys**
Being treated as an individual, with personalised care	Analyses for women born outside the UK are only available for the 2006 and 2010 surveys, for black and minority ethnic (BME) groups:	Recommendations focused on the need for:
Caregivers who are supportive, kind, sensitive, and not rushed		Individualised care for a diverse childbearing population
Care from a small number of staff; knowing the midwives involved in care	Women in these groups were -	Women to be given more choice about place of birth and care provider
Feeling involved in decisions about care and having choices about care options	Less likely to feel spoken to with respect and understanding, and in a way they could understand	More information and opportunity for discussion about care and more involvement for women in decision-making.
Not being left alone in labour	Less likely to feel they had options in care or adequate information	
Being listened to, and spoken to in a way that is understandable	Less likely to describe care providers positively (eg as kind, informative, supportive, sensitive, considerate)	
Being given information and explanations when needed	Less likely to be satisfied with care	
***USA***		
**‘Listening to Mothers’: First national US survey of women’s childbearing experiences**[20,21]**2002**		
n=1583 (1447 online surveys; 136 telephone interviews); numbers of immigrant women not reported		
Overall: For labour and birth, 85-90% reported doctors/midwives and nurses as supportive, understanding and informative, BUT 25% found doctors/midwives rushed and >25% gave less than the highest rating for: information given in a way they could understand;		
**Listening to Mothers II’: Second national US survey of women’s childbearing experiences**[22,23]**2005**		
n=1573 (1373 online surveys; 200 telephone interviews); numbers of immigrant women not reported		
Care for labour and birth from doctors rated as ‘excellent’ by 71% of women; from midwives and nursing staff by 68%		
35% rated the maternity care system as ‘excellent’; 47% as ‘good’; 16% as ‘fair’ or ‘poor’		
**‘Listening to Mothers III’: Third national US survey of women’s childbearing experiences**[24]		
n=2400; 167 immigrant women		
Online survey		
Overall: 80% of women reported their care providers to be ‘completely’ or ‘very trustworthy’ in relation to information about pregnancy and birth		
30% of women said they didn’t ask a question at least once because their care provider seemed rushed		
15% reported that their care provider had used words they did not understand ‘always’ or ‘usually’		
36% rated the maternity care system as ‘excellent’; 47% as ‘good’; 17% as ‘fair’ or ‘poor’		
**Overall findings about what women want: all three surveys**	**Key findings for immigrant women**	**Conclusions and key recommendations: all three surveys**
Being treated with kindness and understanding	No findings have been reported in any of the surveys to date specifically comparing immigrant and non-immigrant women	Key recommendations for care improvements include:
Supportive, unrushed care		Better access for women to effective, safe and appropriate maternity care
Feeling comfortable to ask questions		Improved education of women about their rights to truly informed choice, with full and clear explanations about all aspects of care
Receiving information they needed		Active involvement of women in decision-making
Full and clear explanations understanding what was done and why		
Involvement in decision-making about care		
Non–discriminatory care		
Intervention (only) when needed		

#### Pregnancy care

Women commonly reported problems in pregnancy care with long waiting times, staff not taking time to attend to individual concerns and provide enough information, staff seeming rushed, and lack of continuity of care [3,6,9,12,13,17]. Seeing fewer caregivers during antenatal visits was associated with more positive experiences of care, or was seen as important by women in most studies [6,8,11-13,17]. The need for adequate and consistent information, being treated as an individual, and having effective interaction with caregivers were also commonly reported to be important in shaping positive experiences about pregnancy care [3,8,13,16-18].

#### Intrapartum care

Dissatisfaction with intrapartum care in the population based studies was consistently associated with lack of sufficient information during labour, the perception that caregivers were not kind and understanding, caregivers being unhelpful, and not having an active say in making decisions [4,5,7,15,17,19,21,22],[24].

The nature of women’s interactions with caregivers appears to be a critical factor for women’s experiences at all stages of care. The earliest Australian survey conducted in 1989 revealed a four to sixfold increase in dissatisfaction if women had not received sufficient information from caregivers [5]. Likewise, women who described their caregivers as not being very kind and understanding were four to five times more likely to be dissatisfied with their care; and caregivers regarded as being unhelpful was associated with significant dissatisfaction with intrapartum care [5]. The 2008 national survey in England reported that women were more satisfied with intrapartum care when they received individualised care, enough information and explanations, and were cared for by kind and understanding staff [18]. Involvement in decisions about care and having an ‘active say’ also seem to be consistently important factors associated with more positive experiences of care in labour and birth [5,15,18,19,21,23,24].

#### Postpartum care

Women were less positive about their postpartum care compared with the care they received in pregnancy, or during labour and birth in all three Australian surveys [8-10], in the four UK surveys [16-19] and also in the Swedish study [14].

The factors that seem to be important in women’s experiences of their postpartum care are focused on the attitudes and behaviour of staff: caregivers being sensitive and understanding, providing support and advice, and the helpfulness of that advice and support [10,14-19]. Factors associated with women’s negative experiences of postnatal care included: when their concerns and anxieties were not taken seriously, staff being rushed and too busy to spend time with them, staff not being sensitive and understanding, and not providing enough advice and support about baby care. Another important factor was receiving enough support and advice about women’s own health and recovery [10,15]. In the national Swedish study, content analysis of responses to open-ended questions regarding women’s negative experiences of postpartum hospital care two months and one year after the birth showed that the aspects of care women were most dissatisfied with were: shortages of staff and staff being rushed, staff behaviour, lack of attention to women’s concerns, inadequate support and advice, and lack of sufficient information and explanation regarding baby care and women’s own physical and emotional health after birth [14].

#### Summary of what non-immigrant women want

Drawing on the common themes emerging across the population-based studies from these five countries, we propose the ‘QUICK’ summary, where ‘QUICK’ is a mnemonic that captures the essence of what women want from their maternity care:

Q = Quality care that promotes wellbeing for mothers and babies with a focus on individual needs.

U = Unrushed caregivers with enough time to give information, explanations and support.

I = Involvement in decision-making about care and procedures.

C = Continuity of care with caregivers who get to know and understand women’s individual needs and who communicate effectively.

K = Kindness and respect.

When one or more of these aspects of care was lacking, women were likely to be less happy with their care.

### What do immigrant women want from their maternity care?

#### Findings in the population-based studies

Where data were available for immigrant women in the population-based studies, the key findings have also been included in Table 1. The immigrant women born in countries where English was not the principal language spoken who responded to the three Australian surveys – although unlikely to be representative of all immigrant women, given English language requirements for participation – were less happy with their care than non-immigrant women and more likely to have difficulties with getting the information and support they required [4-10]. In the Canadian [11,12] and Swedish [13,15] studies, similar levels of satisfaction with care were found for immigrant and non-immigrant women, although language issues are acknowledged to have excluded many immigrant women from participation in the Swedish study, and almost one in five immigrant participants in the Canadian study reported not receiving care in a language they could understand [11,12]. Only two of the UK studies [17,19] provided data on immigrant women, with comparisons made for black and minority women without reference to country of birth in the others. Immigrant women of black and minority ethnicity were less likely to feel spoken to with respect and understanding, and in a way they could understand; to feel they had options in care or adequate information; and were less likely to describe care providers positively [17,19]. Findings for immigrant mothers were not reported in the US surveys [20-24] – the third survey did give the numbers of immigrant women participating, but did not report their experiences separately [24].

#### Findings in the studies specific to immigrant women

The findings about what immigrant women value in their maternity care from studies conducted to investigate specific groups of immigrant women’s experiences are summarised in Table 2, and are organised by each receiving country.

**Table 2:** Studies specific to immigrant women’s experiences of maternity care

**Country and study**	**Problems with care as reported by immigrant women**	**Key findings about what immigrant women want**	**Author conclusions and key recommendations**
***AUSTRALIA***			
**Rice & Naksook**[26,27]	Inadequate information about care	Attention to individual needs	Thai women have diverse needs, perceptions and experiences. Women did not receive adequate information about care. An environment needs to be created that acknowledges diversity and meets the needs of individual women.
**1998, 1999**	Difficulties communicating, though some believed care was better in Australia than in Thailand	Support and kindness	
30 Thai women			
In-depth interviews about antenatal, intrapartum and postnatal care	Women felt they were unable to follow traditional customs in hospital.		
**Small** ***et al.*** [28-31]	Communication difficulties	Respectful, understanding caregivers	Vietnamese, Turkish and Filipino women reported similar wants and needs from maternity care as Australian-born women in the companion Survey of Recent Mothers 1994, however these three groups of immigrant women were less likely to experience care that met their needs.
**1998(2), 1999, 2002**			
	Being left alone in labour	Attention to individual needs, not cultural stereotypes	Recommendations included: more attention to the quality of care immigrant women receive and particularly to strategies for overcoming language barriers to effective communication; and better information provision.
**Mothers in a New Country’ (MINC) study**	Not feeling welcomed when came to hospital in labour		
107 Vietnamese women	Experience of discrimination by some staff	Active say in decisions about care	
108 Turkish women	Not enough support about own and infant care postnatally	Information and explanations from staff	
104 Filipino women	Rushed caregivers	Supportive care	
Semi-structured interviews about antenatal, intrapartum and postnatal care	Long waits at antenatal appointments	Recognition of the need to rest and recover post-birth	
	Staff experienced sometimes as unkind or rude and care experienced as culturally stereotyped		
**Tsianakas & Liamputtong**[32,33]	Communication difficulties	Caregivers who show warmth and humanity, and are caring and supportive	Suggestions for care improvement included provision of sufficient information and culturally sensitive services. Health care providers need to attend to individual preferences and circumstances and avoid discrimination.
**2002**			
15 Muslim women from Lebanon, Turkey, Jordan, Egypt, Kuwait, Malaysia, Singapore, Morocco and Pakistan	Perceived stereotyping by caregivers	Female caregivers wherever possible	
In-depth interviews about prenatal testing and antenatal care	Lack of familiarity with services	Good information and explanations, especially about how care is provided and available services	
	Problems with male caregivers	Caregivers sensitive to cultural differences, but able to provide care that responds to individual (not stereotyped) needs	
	Care experienced as discriminatory		
**Tran** ***et al.*** [34]	Difficulties communicating with caregivers	Choice about care options	Authors recommended focus on improvement of service delivery and equity; improving access to interpreter services and bilingual staff; and integrating the biomedical model for maternity services with health beliefs of the diverse cultures.
**2001**			
160 Vietnamese women Focus group discussions, in-depth interviews and survey about care among Vietnamese women who opted for early discharge	Women reported feeling anxious and being fearful when approaching staff for assistance and experiencing discriminition	Adequate advice about self care	
	Disempowerment in culturally unfamiliar hospital surroundings.	Involvement in making decisions about care	
		Supportive caregivers, with enough time to discuss concerns	
		Adequate support and advice about baby care	
**Liamputtong & Watson**[35,36]	Communication difficulties	Adequate information about options for care	Improving communication and access to information identified as essential to ensure women understand all the options available to them.
**2002 and 2006**			
67 Cambodian, Lao and Vietnamese women with experience of childbirth in Australia	Lack of familiarity with care options	Good communication and involvement in decision-making	
In-depth interviews about prenatal testing, and experiences of caesarean birth	Women of ethnic minorities do not have the same access to information and do not understand the implications of services offered to them.	Appropriate help with communication via interpreters and/or support people	
**Chu**[37]	Language difficulties	Caregivers who are friendly and understanding	Authors recommend a focus on empowerment for women and cooperation with community organisations, and service providers to improve cross-cultural communication.
**2005**	Long waiting times		
30 women from Hong Kong, Taiwan and China about childbirth beliefs and care experiences	Insufficient information and advice	Quality in service provision: shorter waiting times	
Semi-structured interviews		Bilingual staff and/or interpreters	
		Supportive after birth care so mother can rest; helpful advice about infant care	
		Adequate information about care options	
**Shafiei** ***et al.*** [38]	Despite care often being seen as better than in Afghanistan, problems identified included:	Unrushed care	Recommendations for care that is more consistently supportive, respectful and caring; strategies to reduce waiting times for antenatal visits, sufficient time for women to ask questions and receive adequate information and explanations, particularly when unfamiliar with how care is provided and when in need of assistance with communication.
**2012**			
		Time and encouragement to ask questions	
40 Afghan women	Long waiting times for antenatal care, rushed staff	Kindness and respect	
Structured telephone interviews about maternity care received when giving birth, with follow-up in-depth face-to-face interviews with 10 women	Problems with communication, lack of interpreting support		
	Insufficient time for adequate information and explanations	Preference for female caregivers	
	At times, unkind, rude staff		
	For some, having male caregivers		
	Problems with hospital food (non-Halal)		
**Hoang** ***et al.*** [39]	Communication difficulties due to lack of English	Supportive care	Authors noted the important role of family and community as in supporting migrant women through their maternity care. Better provision of interpreter services recommended; better social support for women; and reducing cultural barriers through cross-cultural training for health care providers to improve maternity services.
**2009**	Insufficient information offered in other languages	Information and explanations	
10 women from Asia (Vietnam, China, Japan, Korea, Philippines) living in rural Tasmania	Reluctance to express preferences, and make wishes known	Acknowledgment of need for rest and care of mother post-birth	
Semi-structured interviews about care experiences			
***CANADA***			
**Chalmers & Hashi**[40]	Insensitivity of staff to women’s experiences of pain in labour	Involvement in decision-making	Authors highlight need to enhance awareness of cross-cultural practices; address women’s perceptions and needs; use fewer interventions; and provide more respectful treatment. Need also to educate caregivers about traditional female genital cutting.
**2000**			
432 Somali women Structured interviews about experiences of maternity care in Canada in the context of female circumcision	Inappropriate responses to traditional female circumcision (surprise, disgust)	Respectful and sensitive care	
	Felt concerns not listened to		
**Grewal** ***et al.*** [41]	Language difficulties	Family-centred care	Changes in care needed to ensure culturally safe care for immigrant Punjabi women.
**2008**	Lack of familiarity with services and care	Acknowledgement of individual differences in beliefs and preferences	
15 women from Punjab, India In-depth interviews about their perinatal experiences in Canada	Preferences and concerns not acknowledged	Good information about how care is provided and childbirth classes	
		Support for maternal rest after birth	
**Reitmanova & Gustafson**[42]	Inadequate support and inattentive care in labour and postpartum	Adequate information, especially about pain and labour management in labour	Mainstream information and practices designed for Canadian-born women lacks flexibility to meet the needs of immigrant Muslim women. Recommendations included cultural and linguistically appropriate maternity and health information and establishing partnerships with immigrant communities.
**2008**			
	Not enough respect for rest and privacy after birth		
In-depth semi-structured interviews with 6 Muslim women from five countries (not specified) about their experiences of care	Experience of discrimination	Care sensitive to individual needs and beliefs	
	Insensitivity and lack of knowledge on the part of staff about their cultural/religious practices	Appropriate language support and information in community languages	
**Brar** ***et al.*** [43]	Language barriers	Multilingual staff and information/education in community languages, especially about available services and care	Recommendations of authors include the need for multilingual staff and provision of educational materials in a variety of formats.
**2009**			
	Unfamiliarity with care provided	Supportive care and adequate help with infant care	
Structured interviews with 30 south Asian and 30 Canadian-born women about maternity care and perceived barriers	Lack of explanations for tests and procedures	Women caregivers	
	Lack of assistance with baby care after birth		
***SWEDEN***			
**Essen** ***et al.*** [44]	Lack of knowledge among staff for handling traditional female circumcision	Good monitoring of health of mother and checks during pregnancy, and of infant after birth	Authors conclude that health providers need to improve their knowledge about female circumcision and also provide culturally sensitive perinatal surveillance in order to address women’s concerns and any cultural misconceptions about pregnancy and birth.
**2000**			
15 Somali women	Not enough emotional support	Kind, attentive care; sensitivity to individual needs, especially care for female circumcision	
In-depth interviews about childbirth and experiences of care in Sweden	Fear of caesarean section		
**Berggren** ***et al.*** [45]	Although pleased with high standard of clinical care, made to feel ashamed of their traditional female circumcision by some staff	Sensitive and understanding care	Authors recommend culturally adjusted care and providing systematic education about female circumcision.
**2006**			
21 women from Somalia, Eritrea and Sudan	Requests not dealt with sensitively	Good communication	
Exploratory interviews about maternity care in the context of traditional female circumcision	Language difficulties	Attention to individual needs	
	Felt unable to follow certain cultural beliefs/traditions		
***UNITED KINGDOM***			
**Woollett & Dosanjh-Matwala**[46,47]	Communication difficulties	Sensitive, respectful care attentive to individual needs and concerns	Authors discuss issues and implications of differences between women and services in what is considered ‘normal’ maternal behaviour and the need to improve the quality of care to immigrant women, especially to attend to individual and cultural diversity.
**1990**			
	Long waiting times	Careful monitoring of health of mother, and fetus/infant	
32 women, 19 of whom were immigrants (countries not specified: India, Pakistan and Bangladesh??).	Staff rushed, no time for discussion	Good explanations and information about care and tests; careful physical checks	
Women spoke Hindi, Punjabi and Urdu and/or English Semi-structured interviews	Lack of support from staff, especially postnatally when women most of all wanted to rest	Good support for rest and care of infant in hospital after birth	
**McCourt & Pierce**[48]	Communication/language difficulties	Good communication and information about options for care	Authors note that minority ethnic women in fact shared similar values and had expectations of services similar to the wider population, but that conventional services did not provide minority ethnic women with high quality of maternity care. The authors suggest this is related to the institutional organisation of care which needs to become more focused on addressing *all* women’s individual needs.
**2000**			
	Inadequate information about care options	Friendly, kind staff	
20 ‘minority ethnic’ women interviewed, including 6 Somali women about experiences with maternity care (half caseload and half standard care)	Staff rude or off-hand (standard care)	Good access to interpreting services when needed	
Qualitative interviews	Concerns not listened to	Attention to individual concerns	
	Not enough support for rest after birth	Primary care provider who gets to know each woman and her needs	
		Acknowledgement of need for rest and support after birth	
**Davies & Bath**[49]	Poor communication with staff	Good care and adequate information about options for care	Key underlying problem considered to be poor communication between non-English speaking Somali women and health workers. This needs to be addressed with better use of interpreters and more individualised care.
**2001**	Limited use of interpreters		
13 Somali women:	Prejudiced attitudes of staff	Attention to specific individual needs	
Focus group and structured interviews about ‘maternity information concerns’.	Lack of information	Supportive care and rest after birth	
**Harper Bulman & McCourt**[50]	Poor communication and inadequate provision of interpreting services led to needs not being met	Kind and attentive staff	Need for better integrated and more appropriately used interpreting services that enable greater continuity for women. Advocacy or link-worker schemes may also be appropriate.
**2002**			
12 Somali women:	Not enough information and discussions about important topics, such as managing pain	Better interpreting services	
Six Individual in-depth interviews and two focus groups	Stereotyping and racism from staff	Staff who understand when interpreters are needed	
	Lack of understanding of cultural differences		
**Jayaweera** ***et al.*** [51]	Language difficulties (but assisted when interpreters available)	Good use of interpreters to assist communication and provision of information	Considerations need to be made for social and economic circumstances of migrant families.
**2005**			
	Reduced care options when English lacking		
9 Bangladeshi women (8 immigrants)			
Semi-structured interviews about childbirth experiences and needs			
***USA***			
**Herrel** ***et al.*** [52]	Experiences of discrimination in interactions with nurses believed to be due to skin colour and/or lack of English	Supportive, non-discriminatory care with a known care provider	Need culturally appropriate health education materials on labour and delivery for the Somali refugee community. Health care teams need to receive training on Somali culture, traditions and values and Somali women’s expectations.
**2004**			
14 Somali women	Inadequate information about pain relief and side effects	Full explanations	
Two focus groups with 20-item interview guide, facilitated by Somali-speaking group moderator	Poor explanations (eg for caesarean birth, which women feared)	Hospital tour with language support	
	Communication problems and concern about the competence of interpreters.	Education for partners to familiarise them with women’s needs for pregnancy and birth	
		Information about services in accessible language & format (eg videos)	
**Jambunathan and Stewart**[53]	Communication problems with health care providers	Preference for minimal intervention in pregnancy and birth	Health care providers need to better understand Hmong women, eg when touching and communicating with women and informing them about hospitalisation and medical procedures.
**1995**			
	Miscarriage feared if touched by doctors and nurses which resulted in delayed prenatal visits	Understanding from care providers about women’s own experiences and concerns	
52 Hmong women			
Semi-structured interviews conducted 4-6 months after birth	Wary about interventions and procedures for labour and birth		
**Lazarus and Phillipson**[54]	Long clinic waits	Reduced waiting times	Few differences reported: Puerto Rican and ‘white’ women wanted the same things from care.
**1990**	Insufficient time at appointments	More time at appointments	
27 Puerto Rican women (17 immigrant, 10 born in the US) and 26 indigent ‘white’ women; and 150 observations of clinical interactions	Poor communication and explanations	Known care providers	
	Many different physicians for prenatal care: contradictory advice, lack of familiarity with woman’s concerns and circumstances	Sound information and explanations that can be understood	
Qualitative interviews about prenatal care conducted prospectively from early pregnancy, combined with anthropological observations of prenatal care interactions		Better communication about care (not just because of language problems)	
**Shaffer**[55]	Problems with communication due to language barriers	Being able to communicate with health care providers in own language	Authors recommend culturally appropriate health care to meet Hispanic migrant women’s needs.
**2002**			
46 Hispanic migrant women Qualitative interviews during pregnancy exploring factors influencing access to prenatal care		Culturally appropriate health care	

Table 2 shows that the findings from these studies are not only quite consistent across immigrant groups originating from very different cultures and countries, but also that the ‘QUICK’ summary elements found in the population studies, appear also to be central in the accounts of immigrant women from these immigrant-specific studies, again regardless of women’s country or culture of origin, or of the country to which they had migrated.

However, additional challenges associated with negative impacts on women’s experiences of care emerge from the studies of immigrant women. First, language difficulties clearly hamper good communication and understanding between immigrant women and their caregivers when women are not fluent in the language of the receiving country. Communication difficulties were identified as a key problem in almost all the immigrant studies [25-29,32-35,38-45,47-49,51,55]. Lack of information in community languages and insufficient access to interpreters when needed were also commonly reported and a few studies noted that even when interpreters were available, women did not always feel that they were competent [25,45,47]. Lack of familiarity with how care is provided or not receiving adequate information about what options for care exist, were also common problems for immigrant women [26,28-32,35-38,41,48,50,51]. Several studies also reported immigrant women feeling they were not welcomed, or were made to feel anxious, when they came to hospital in labour [28-31,34,37].

Despite evidence that immigrant women want to be involved in decisions about their care, [28-31,39-41] some studies found that immigrant women were at times reluctant to make their wishes known [39,41]. Experiences of discrimination, and/or cultural stereotyping were also commonly reported in the immigrant studies from all five countries [28-32,40,42,44,45,48,50,52]. Studies of Somali immigrants in Canada, Sweden and the UK also found that women felt staff were insensitive to their experiences of pain in labour and responded inappropriately to traditional female genital cutting, demonstrating a lack of knowledge about this issue [40,44,45,50].

Some studies noted particular cultural issues that immigrant women felt were not well understood during their maternity care and about which they desired more understanding from their caregivers. One US study of Hmong women described women's fears of being touched by doctors and nurses because of beliefs about the causes of miscarriage [53]. Some studies reported women's preference for female caregivers, [28-32,43] with Muslim women in particular expressing this preference. It is worth noting however that this question is rarely asked in studies of non-immigrant, or non-minority women, so whether immigrant women are more likely to prefer female caregivers than non-immigrant women is not readily known. Several Australian studies found that women sometimes found it difficult to follow traditional cultural practices in hospital (for example food preferences, not showering after birth), and women reported that they were rarely asked by caregivers about their postnatal practice preferences [26,27,31,37,39].

Interestingly though, lack of attention to cultural issues or restrictions on traditional cultural practices by caregivers were not the principal focus of women's descriptions of negative aspects of the maternity care they received post migration. Communication problems and discriminatory or negative caregiver attitudes appear to be the more critical areas of concern reported by women in the studies reviewed here, just as immigrant women's positive experiences of care centred around appreciation of being treated with kindness and respect and having their individual concerns addressed competently and sympathetically.

Two published systematic reviews of studies of immigrant women’s experiences of childbirth and maternity care broadly support the findings about immigrant women’s experiences from our five included countries [57,58]. The first is a recent systematic review which included 16 qualitative studies from six European countries (Greece, Ireland, Norway, Sweden, Switzerland and the UK). It aimed to investigate immigrant women’s needs and experiences of pregnancy and childbirth and found as we did, that good communication and information, an understanding of how care operates in their new homeland, caregivers who are respectful, non-discriminatory and kind, and achieving a safe pregnancy and birth are key aspects of what immigrant women wanted from their maternity care [57]. The second review [58] included 40 qualitative studies from Australia, Canada, Denmark, Ireland, Israel, Japan, Norway, South Africa, Sweden, and the USA. Aiming to explore aspects of intercultural caring from immigrant women’s perspectives of their maternity care, the review concludes that addressing communication problems, providing continuity of care, addressing racism and discrimination and providing flexibility in care to accommodate individual and cultural diversity are likely to enhance immigrant women’s experiences of maternity care. What the current review additionally offers is a comparison with non-immigrant women, previously missing in the literature.

### Strengths and limitations

This review has drawn together the available population-based studies of women’s experiences of maternity care in order to assess what is known about immigrant compared with non-immigrant women’s experiences. As immigrant women have often been under-represented in population-based research, we supplemented our review of these studies with the findings from studies focused on specific groups of immigrant women in each of the countries where population-based studies were identified. This is both a strength, and a limitation. It could be said that we are not comparing like with like, and that is true. Most of the specific immigrant studies are small and qualitative in design and the representativeness of the immigrant participants cannot be ascertained. On the other hand, synthesising the evidence from a range of study types for immigrant women, in an area where assembling representative samples is particularly difficult, has proved informative, particularly given the consistency that has emerged in the findings from both the population-based and the qualitative studies. Examining studies drawn from the same receiving countries is also a strength of this review. Had factors associated with different maternity care systems been important in shaping women’s experiences of care, then this should have become apparent in comparisons of women’s experiences in the different countries. It is significant that at least in relation to care in Australia, Canada, Sweden, the UK and the United States of America, women identify the same problems with care and articulate very similar wishes in relation to what they want from care when giving birth. We are not aware of other reviews that have as yet attempted to directly compare immigrant and non-immigrant women’s experiences of care within and across countries, as we have done here.

Finally, this review is limited by the studies that have been conducted to date. Globally, relatively few countries have undertaken population-based studies of women’s experiences of their maternity care. Of these, only the Canadian study has used a multi-language strategy in an attempt to address the under-representativeness of immigrant women in population studies, and the Australian research involved a companion study of three immigrant groups [28-31] in tandem with one of the three population surveys [4-8] undertaken there. It is also worth noting that the recent waves of migration between countries in the European Union and of refugee and asylum-seeking arrivals are not yet well represented in studies of women’s experiences of maternity care.

### Summary of the key findings

This review has found that immigrant and non-immigrant women appear to have very similar ideas about what they want from their maternity care, notwithstanding the diversity of countries and cultures of origin of the women represented in the reviewed studies. In regard to women’s overall ratings of their maternity care however, immigrant women commonly gave poorer ratings of the care they received compared with non-immigrant women, and a range of additional challenges they faced tended to have negative impacts on their experiences of care. These chiefly included: communication difficulties due to language problems, lack of familiarity with how care was provided and experiences of discrimination.

Authors of the studies of immigrant women often recommended the need for more culturally sensitive care, with cultural competency training for maternity services staff seen as a means to this end. While in some studies immigrant women did comment on staff not understanding their cultural beliefs and practices, a careful examination of what women most commonly wanted – as shown in Table 2 – demonstrates that women themselves were focused more on the need for respectful care that was attentive to their individual needs, on assistance with communication difficulties and on receiving better information about how care is provided in their new country. Women in more than one study commented that staff cannot possibly ‘know’ every culture. Moreover, cultural beliefs and practices are not static phenomena, with considerable diversity among women from within any one culture with regard to adherence to particular traditions or beliefs, so that encouraging staff to ask all women about their childbirth preferences and beliefs is likely both to be more achievable, and also to result in more responsive care for all women, immigrant and non-immigrant alike.

Notably in this review, women from a range of immigrant backgrounds in studies from all five receiving countries, reported problems with discrimination or prejudice in their experiences of care. If services are to take seriously what immigrant women say they want, then perhaps what is most needed to improve care is an enhanced focus on promoting equity and non-discriminatory attitudes in care provision, along with strategies aimed at improving communication (including training in working effectively with interpreters), and better recognition of the need to familiarise immigrant women with how maternity care is provided, so that they can more actively participate in decisions about their care and feel less anxious and disempowered about giving birth in their new country.

## Conclusion

What this review has revealed is that improvements in immigrant women’s often poorer ratings of care will only come if more attention is paid to addressing the additional challenges they face due to language difficulties, lack of familiarity with care systems and at times, exposure to discriminatory attitudes and poorer quality care. Proper recognition of these extra challenges is required in the provision of care. In addition, maternity staff need to be supported – with time, resources and training – to enable them to provide appropriate and non-discriminatory care to immigrant women, in accord with published declarations and standards of quality care for immigrant populations [59,60]. More inclusive approaches to enable the involvement of immigrant women in future population-based studies of women’s experiences of maternity care would also ensure that care improvements for immigrant women can be appropriately evaluated over time.

## Competing interests

The authors declare that they have no competing interests.

## Authors’ contributions

RS, CR, DK, AG and MH conceived the project; MR conducted the searches; MR, TS and RS reviewed the studies for inclusion; MR developed the first draft of the tables of studies and these were checked and modified by RS and TS, and subsequently by all authors. RS, MR, TS and CR were involved in drafting the manuscript and all authors (RS, CR, MR, TS, DK, CMcC, MH and AG) contributed to revising it critically for intellectual content and all approved the final manuscript.

## Pre-publication history

The pre-publication history for this paper can be accessed here:

http://www.biomedcentral.com/1471-2393/14/152/prepub

## Supplementary Material

Additional file 1 PRISMA checklist, including example search strategy. Click here for file
